# Hybrid feature-based machine vision method for objective evaluation of textile pilling and fuzzing

**DOI:** 10.1371/journal.pone.0329814

**Published:** 2025-09-03

**Authors:** Qingchun Jiao, Zifan Qian, Yue Dong, Bo He

**Affiliations:** 1 School of Automation and Electrical Engineering, Zhejiang University of Science and Technology, Hangzhou, China; 2 Zhejiang Institute of Quality Science, Hangzhou, China; Kafkas University: Kafkas Universitesi, TÜRKIYE

## Abstract

The degree of pilling and fuzzing in textile fabrics is a crucial indicator of textile product quality. Current evaluation methods predominantly rely on subjective judgments, leading to issues such as rating errors and inefficiency. To achieve objective assessment of pilling and fuzzing grades, this study proposes a Hybrid Feature-Based Machine Vision Method for Objective Evaluation of Textile Pilling and Fuzzing. The method incorporates a Hybrid Feature-based Depthwise Separable Attention Network for Objective Evaluation of Textile Pilling and Fuzzing (HDAN-PF), which effectively extracts and fuses frequency and Space domain features. A Channel Attention mechanism enhances the model’s ability to capture subtle features, while Depthwise Separable Convolutions reduce computational complexity, improving evaluation speed while maintaining high accuracy.The model size is approximately 327.37 MB with a total parameter count of 135,115,512. Experimental results demonstrate that the proposed method achieves a classification accuracy of 96.26% on diverse fabric images, showcasing robust generalization and practical utility.By leveraging this machine vision approach, the proposed method offers a transformative solution for achieving objective, consistent, and efficient assessments of pilling and fuzzing grades, advancing textile quality evaluation practices.

## Introduction

Textiles, as a primary component of many products, directly impact the quality and comfort of the final goods [1]. With rising living standards, consumer expectations for fabric quality and tactile experience have also become more refined [2]. However, pilling and fuzzing frequently occur on fabric surfaces due to fiber friction and structural changes, forming particle clusters that detract from both appearance and performance, including hand feel and abrasion resistance [3,4]. Complaints regarding pilling and fuzzing constitute approximately 29.51% of textile quality issues [5], underscoring the importance of these characteristics in quality assessment.

Currently, pilling level detection in textiles is primarily conducted through manual visual inspection, which, due to the diversity in fabric types and pilling degrees, demands substantial expertise from inspectors [6]. Prolonged visual inspection often leads to subjective fatigue, introducing variability in inspection results. To ensure consistency, multiple experts are typically involved in joint reviews, which is both time-intensive and costly [7]. This context highlights the need for an objective, automated evaluation system.This context highlights the need for an objective, automated evaluation system, as a textile pilling and fuzzing quality evaluation system can automate fabric quality assessment, reduce human detection errors, and improve detection efficiency. It enhances product consistency and promotes the industry’s transition toward smart manufacturing, automation, and digitalization.

In response, researchers have explored computer image processing technology to achieve more objective and efficient fabric pilling evaluations. Current methods include Space domain techniques that analyze color or grayscale information for pilling detection and frequency domain techniques that use frequency information to distinguish pills from fabric [8]. However, each approach has limitations: Space domain methods are vulnerable to image noise, while frequency domain methods are sensitive to image size and morphology [9].

To overcome these limitations, this paper presents a Hybrid Feature-Based Machine Vision Method for Objective Evaluation of Textile Pilling and Fuzzing. This approach integrates frequency and Space domain characteristics, leveraging preprocessed grayscale images to capture fabric texture features. Retaining grayscale images helps mitigate noise effects when processing textured patterns, enhancing classification accuracy.We further developed a Hybrid Feature-based Depthwise Separable Attention Network for Objective Evaluation of Textile Pilling and Fuzzing (HDAN-PF), which is based on deep convolutional neural networks. We optimized the number of inception modules and convolution kernel sizes to achieve an optimal balance between network depth, generalization capability, and computational cost. By coordinating the application of the attention mechanism and Depthwise Separable Convolution (DSC) technology, we not only optimized the model structure but also improved computational efficiency. The main contributions of this paper are as follows:

(1) We propose a Hybrid Feature-Based Machine Vision Method that effectively combines frequency domain, Space domain, and grayscale image features for objective evaluation of pilling and fuzzing.
(2) We design the HDAN-PF, incorporating the Coordinate Attention (CA) mechanism to enhance the detection accuracy of pilled and fuzzed areas.
(3) We utilize DSC technology to reduce computational complexity and improve the model’s operational speed.
(4) We construct a diverse dataset covering various fabric types, textures, colors, and patterns to validate the model’s generalization across different samples.

## Related work

### Current status of objective evaluation of fabric pilling and fuzzing performance

To enhance textile quality and user experience, accurately evaluating the pilling and fuzzing performance of fabrics is of great significance. This not only affects the appearance retention and durability of products during use but also serves as an important reference for textile companies in product research and development and quality control. Various testing methods simulating real-world wearing or washing environments have been established in the industry to detect pilling and fuzzing phenomena caused by friction on fabric surfaces [10]. Although there are differences in the standard systems across countries, with the United States commonly using American Society for Testing and Materials (ASTM) standards, Europe relying on International Organization for Standardization (ISO) international standards, and China following Guobiao/Tuijian (GB/T) national standards or enterprise standards, these standards share fundamentally similar principles in terms of testing procedures and evaluation principles [11]. However, due to differences in testing methods and operational conditions, rating results in practical testing can still vary, further increasing the subjectivity and uncertainty of manual evaluations.

Currently, fabric pilling and fuzzing grade assessments still primarily rely on manual visual inspection. Although various standards clearly define the grading process and standard reference images, and require testing personnel to compare the test samples with the reference images and assess the grade based on descriptive criteria to ensure the consistency and standardization of the evaluation, many issues remain in practical applications [12]. On one hand, the grading process is cumbersome and inefficient. On the other hand, the results heavily depend on the experience level and visual sensitivity of the evaluator, making them susceptible to subjective judgment, visual fatigue, and the diversity of fabric types, leading to poor consistency and repeatability [13]. Particularly in large-scale sample testing or industrial applications requiring high grading precision, traditional manual grading methods are increasingly inadequate for modern quality control needs [14]. Therefore, developing an efficient, objective, and robust automatic grading method has become an important research direction.

With the rapid advancement of computer vision and artificial intelligence, image analysis-based objective evaluation methods have emerged as a mainstream research focus. Within the Space domain, objective rating methods based on image analysis and processing primarily include threshold processing and image reconstruction. For example, Sekulska-Nalewajko et al. [15] proposed a method using Optical Coherence Tomography imaging to analyze fabric texture characteristics and objectively assess the degree of pilling and fuzzing, achieving precise identification even in the early stages of pilling. Hassan [16] combined image analysis technology with artificial neural network models, extracting and calculating features from specific digital images, then classifying the fabric pilling grades accurately according to the obtained pilling feature vectors within a pre-trained neural network following ASTM standards. Liu et al. [17] used a mobile camera system and stereo vision algorithms to achieve three-dimensional reconstruction of fabric surfaces, extracting depth information to assist in the parameterization of pilling features, thereby enhancing the objectivity and accuracy of evaluations. Guan et al. [18] proposed a deep learning method combining saliency preprocessing with an improved ResNet 34 network model. By constructing a larger and more diverse fabric pilling and fuzzing dataset, they effectively enhanced the accuracy of fabric pilling grade assessments, achieving an average accuracy rate of 93.88%. However, threshold processing algorithms in the Space domain are susceptible to interference from fabric texture, color, and patterns, potentially leading to omission or introduction of invalid pilling information, thus reducing detection accuracy. Moreover, although three-dimensional image reconstruction methods can improve precision, they require higher computational resources and costs, posing significant challenges in practical applications.

Within the frequency domain, objective rating methods primarily involve transforming signals or data mathematically into the frequency domain and selecting appropriate filters to extract pilling information. For instance, Wang et al. [19] used Fourier transforms and wavelet transforms to obtain Gaussian distributions of pilling frequencies and analyzed their impact on the pilling performance of polyester-cotton blended woven fabrics. Zhan et al. [20] proposes an innovative objective evaluation method for fabric pilling by integrating image analysis techniques in the frequency domain with a deep learning algorithm, achieving a classification accuracy of 94.2%. Although frequency domain evaluation methods perform well in separating pilling information, specific adjustments to filter parameters are needed for different fabric types, limiting their performance in classifying different fabric materials.

For example, Huang et al. [21] proposed a homomorphic filtering-based method for assessing the pilling and fuzzing grades of colored fabrics, achieving a comprehensive grading accuracy of 91.6%. Although this method integrates both space and frequency domain features, it loses the fabric’s texture information after the inverse transformation, thus performing poorly in classifying images with noise.

Compared to these methods, this paper introduces a method that simultaneously utilizes Space and frequency domain features input into a neural network for fabric pilling grade assessment.By comprehensively capturing details and global patterns, the method proposed here overcomes the limitations of existing methods by reducing texture interference in the Space domain and eliminating the need for filter parameter adjustments in the frequency domain. This approach enhances the model’s recognition accuracy and robustness under complex backgrounds, improving learning capability and processing efficiency, and achieving high accuracy and efficiency in fabric pilling assessment.

### Machine vision

Machine vision is a technology that enables perception and analysis capabilities in machines by utilizing computer and image processing techniques. It involves acquiring images or videos through hardware devices such as cameras and sensors, followed by analysis using image processing algorithms to perform tasks like object recognition, measurement, detection, and control. Early machine vision methods primarily relied on traditional image processing algorithms, such as edge detection and shape matching [22]. With technological advancements, machine learning and pattern recognition have been increasingly integrated into machine vision [23]. In particular, the rapid development of deep learning in recent years has expanded the application of convolutional neural networks (CNNs) in this field, significantly enhancing the performance and adaptability of machine vision systems [24,25]. These systems can now handle more complex scenarios and achieve high-precision recognition tasks. The applications of machine vision are extensive, spanning industrial automation, medical image analysis, agricultural product inspection, and more [26,27]. In industrial automation and quality control, machine vision-based techniques have become indispensable in intelligent manufacturing systems, enabling real-time monitoring, precision detection, and automated control to meet the high-precision and automation demands of Industry 4.0 [28].

Recently, DSC and CA mechanisms have become integral to machine vision, demonstrating significant advantages in image processing tasks. DSC, by decomposing standard convolution operations, substantially reduce computational costs and improve processing speed and efficiency, making them particularly suitable for real-time processing of large-scale visual data. For example, Liu et al. [29] developed an efficient lightweight network for defect detection in polarizers (DDN) by employing DSC. This approach notably reduced parameters and computational load while enhancing image processing speed and accuracy, fulfilling the real-time requirements of online detection systems. Zhang et al. [30] further optimized the CURI-YOLOv7 model using DSC, achieving a significant reduction in computational complexity and model size while maintaining a high detection accuracy of 90.34% mAP. This advancement enabled rapid detection of single citrus trees in Unmanned Aerial Vehicle (UAV) remote sensing images, making it suitable for real-time applications on embedded devices.

CA mechanisms, by focusing on key feature regions in images, have significantly improved model robustness and accuracy, particularly in handling complex backgrounds and multiple objects [31]. For instance, Li et al. [32] proposed CCAFusion, integrating cross-modal CA mechanisms to effectively combine complementary features of infrared and visible-light images. This method enhanced image fusion quality and performance in advanced vision tasks such as salient object detection.

The combined application of DSC and attention mechanisms has also demonstrated superior performance. Li et al. [33] introduced an enhanced 3D convolutional neural network (3DCNN-AM-DSC) that incorporates both techniques, achieving a 91.77% reduction in training time while maintaining excellent classification performance in hyperspectral remote sensing image classification. These findings highlight that the synergy of these technologies not only optimizes model performance but also significantly reduces computational overhead.

Although studies on quality inspection of textile pilling and fuzzing are relatively scarce, the successful applications of DSC and attention mechanisms in industrial inspection and remote sensing indicate substantial potential for machine vision in this domain. DSC, with their efficient computational capabilities, can process textile sample data rapidly, while attention mechanisms enable detailed focus on critical surface areas, improving the detection of pilling and fuzzing features.

Leveraging high-precision cameras and advanced image processing algorithms, machine vision can capture subtle defects on textile surfaces. The integration of these techniques further optimizes feature extraction and analysis, enhancing detection efficiency and quality control. As these technologies mature, breakthroughs in textile pilling and fuzzing inspection are expected, reducing human error and ensuring stable and consistent detection outcomes. This will provide the textile industry with efficient and intelligent quality inspection solutions, driving its progress toward automation and intelligence.

## System architecture

### System composition

**Fig 1:**
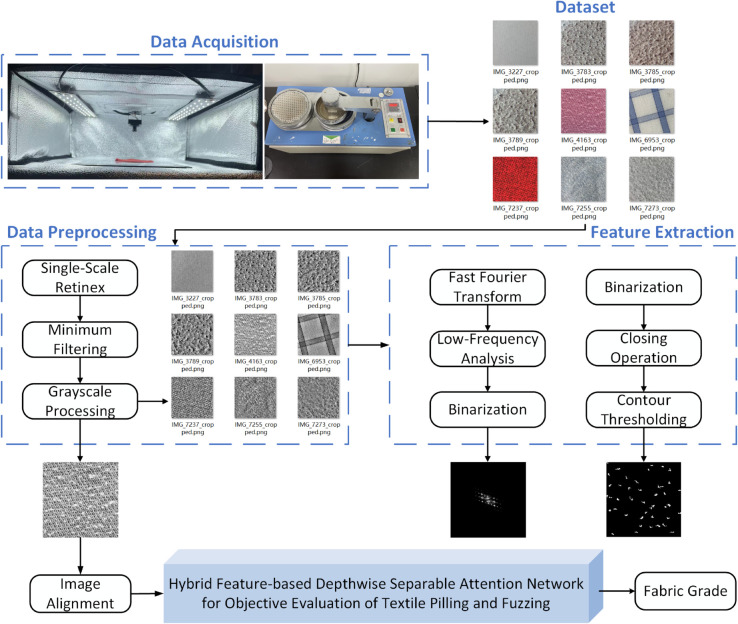
Hybrid feature-based machine vision system for textile pilling and fuzzing performance evaluation. This system consists of five main components. The Data Acquisition section presents the machines and tools used for pilling and fuzzing tests. The Dataset section provides a sample of the collected images. After data preprocessing and feature extraction, grayscale, Space domain, and frequency domain feature maps are generated. These feature maps are then input into the HDAN-PF, which classifies the images to determine the fabric’s pilling and fuzzing grade.

To enable efficient assessment of pilling and fuzzing grades in textile fabrics, this study develops a Hybrid Feature-Based Machine Vision System for Textile Pilling and Fuzzing Evaluation. The system encompasses data acquisition, preprocessing, feature extraction, and application of the HDAN-PF model, providing a systematic and objective approach for textile quality evaluation, as shown in Fig 1. By integrating multiple feature types, the system aims to improve evaluation accuracy and reliability.

### Workflow

The system workflow involves the following steps: First, images of worn fabric areas are captured via an image acquisition device. These images then undergo preprocessing, including grayscale conversion, minimum filtering for noise reduction, and Single-Scale Retinex for brightness and contrast enhancement. Next, a Fast Fourier Transform (FFT) converts the images to the frequency domain, allowing extraction of low-frequency components associated with pilling and fuzzing characteristics. Concurrently, the preprocessed images are binarized and processed with a Closing Operation to remove noise and enhance Space domain feature extraction. The grayscale image, serving as a comprehensive feature map, along with the Space and frequency domain feature maps, are then input into the HDAN-PF model to classify pilling and fuzzing into five grades.

To ensure system robustness and effectiveness, key factors were carefully considered during the workflow design. For example, the choice of image acquisition device affects data quality, while the selection of filtering and enhancement algorithms during preprocessing impacts feature extraction accuracy. Integrating frequency and Space domain features is critical for accurate classification; hence, feature combination methods were optimized in the network architecture design. This systematic workflow aims to provide a reliable and scientific method for assessing fabric pilling and fuzzing performance.

To ensure system robustness and effectiveness, key factors were carefully considered during the workflow design. For example, the choice of image acquisition device affects data quality, while the selection of filtering and enhancement algorithms during preprocessing impacts feature extraction accuracy. Integrating frequency and Space domain features is critical for accurate classification; hence, feature combination methods were optimized in the network architecture design. This systematic workflow aims to provide a reliable and scientific method for assessing fabric pilling and fuzzing performance.

## HDAN-PF

This study proposes the HDAN-PF, a network that integrates Space, frequency, and grayscale features to achieve high performance and efficiency in evaluating fabric pilling and fuzzing grades. The core innovation of the model lies in its balance between depth, generalization, and computational efficiency, strengthened by the integration of the CA mechanism and DSC technology, which enhance robustness and reduce computational demands.

**Fig 2:**
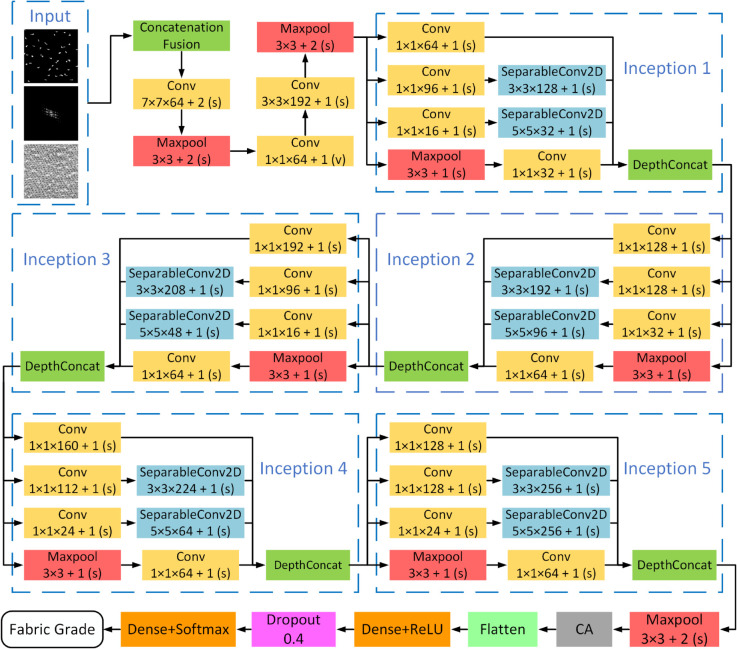
HDAN-PF. Illustrates the main architecture of the HDAN-PF network. Through Concatenation Fusion, multiple features are input into the model. A 5-layer Inception, DSC, and CA are employed to optimize the network, ultimately determining the fabric’s pilling and fuzzing grade.

As shown in Fig 2, the overall network architecture is based on deep CNNs, achieving an optimal balance between network depth, generalization, and computational cost by adjusting the number of modules and convolutional kernel sizes.Unlike conventional models such as ResNet and DenseNet that rely mainly on deep hierarchical spatial feature extraction, HDAN-PF fuses grayscale, spatial domain, and frequency domain features to form a hybrid representation, improving sensitivity to subtle surface variations. The use of DSC enhances computational efficiency, while the CA mechanism enables more precise localization of fine-grained texture regions critical for pilling and fuzzing assessment. These architectural differences highlight the task-specific design of HDAN-PF and its advantage in textile quality evaluation scenarios.

### Inception module

**Fig 3:**
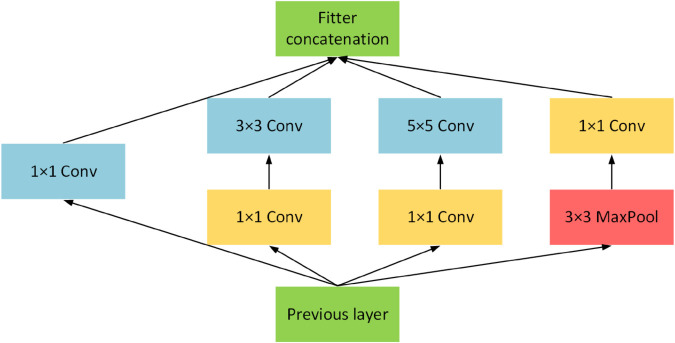
Inception module. The diagram illustrates the basic structure of an Inception module, demonstrating how convolutional kernels of varying sizes (1x1, 3x3, and 5x5) are integrated alongside a max-pooling layer to work in concert. This design allows for the simultaneous processing of multi-scale features within a single layer.

The Inception module is inspired by the “network-in-network” concept. Its primary objective is to expand the network’s depth and width by embedding multiple smaller network units while controlling the parameter scale and computational cost. This design integrates convolutional kernels of various sizes (such as 1x1, 3x3, and 5x5) and max-pooling operations, enabling parallel extraction and fusion of multi-scale features to create a hierarchical, dimensionally rich feature representation. As shown in Fig 3, the lightweight design of the Inception module reduces parameter count, simplifies training, and improves computational efficiency. When processing high-resolution fabric images, the 1x1 convolution layer and global average pooling further decrease computational load while preserving detailed information, ensuring model efficiency and comprehensive feature capture.

In fabric pilling and fuzzing assessments, the Inception module’s multi-scale processing capability offers notable advantages. It captures variations from small fiber entanglements to larger fuzzball structures, ensuring that both high-frequency details and low-frequency patterns are effectively extracted by convolutional kernels of different sizes. This enhances the model’s performance in fabric pilling and fuzzing assessments and provides a new approach for efficiently handling frequency and Space domain features in fabric quality control.

### CA mechanism

In deep learning-based image recognition, attention mechanisms allow models to focus on critical image regions, improving classification accuracy and efficiency. For fabric pilling and fuzzing assessments, the CA mechanism strengthens feature representation by combining channel and Space attention, allowing the model to identify and prioritize regions associated with pilling and fuzzing while ignoring irrelevant background information.

**Fig 4:**
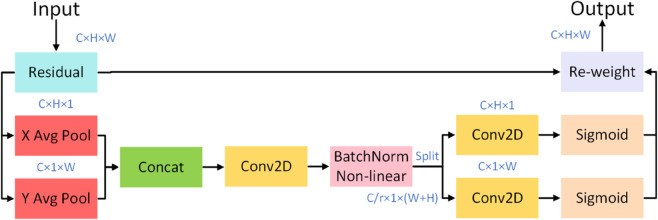
CA Mechanism flow. The diagram illustrates the workflow of the CA mechanism. It generates attention vectors for width and height using 1D convolutions, capturing Space information. These vectors are then multiplied to form a comprehensive attention map, highlighting key areas. Finally, the attention map is applied to the input feature map to enhance features.

The CA mechanism involves several steps: First, the input feature map is decomposed along the horizontal and vertical axes, with global average pooling capturing global information in each direction to detect long-range dependencies in the feature map. The decomposed features are then encoded to enhance directional feature representation, followed by a pointwise (1x1) convolution that maps the encoded features back to the original feature map size, reconstructing attention maps along both axes. Finally, the original feature map and attention maps are fused through weighted summation, creating enhanced feature representations. As shown in Fig 4, this process ensures the model can accurately capture prominent regions related to pilling in the image.

In fabric pilling and fuzzing grade assessments, the CA mechanism effectively captures long-range dependencies and preserves key Space information, enhancing the model’s ability to differentiate between various pilling and fuzzing levels. By strengthening feature representation, the CA mechanism significantly improves the model’s perception of fine surface details, optimizing the evaluation process with high accuracy and consistency. This mechanism thus demonstrates strong potential for enhancing model performance, efficiency, and reliability, supporting deep learning applications in fabric quality control.

### DSC

**Fig 5:**
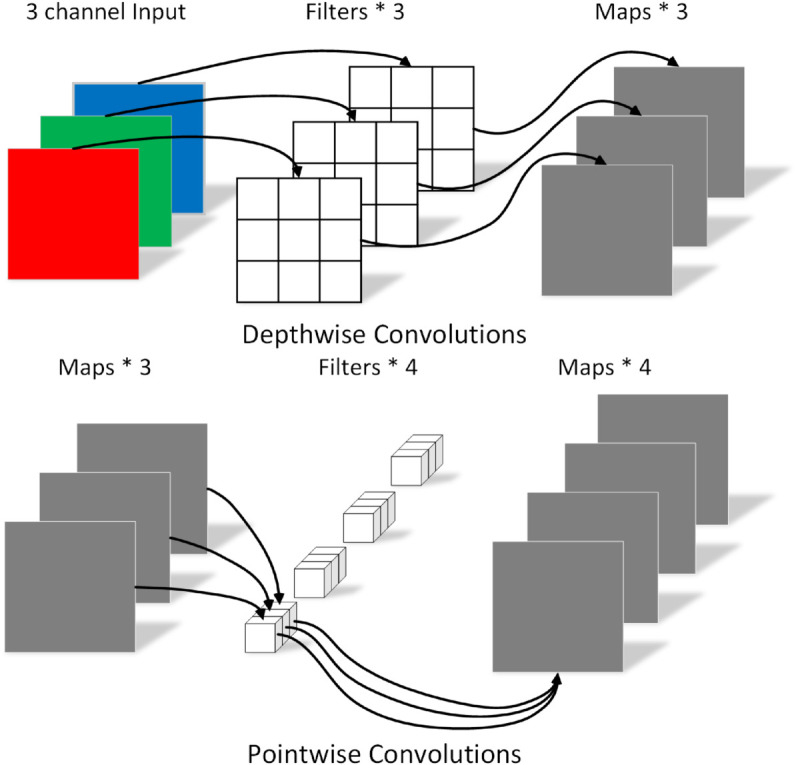
DSC. The diagram shows the DSC workflow: depthwise convolution applies kernels to each input channel independently, followed by pointwise convolution using 1x1 kernels to combine feature maps.

DSC is an efficient convolutional strategy that decomposes the convolution operation into two steps: depthwise convolution and pointwise convolution. This reduces computational requirements to about *k*/1 of traditional convolutions (where *k* is the kernel size), lowering model parameters, increasing inference speed, and mitigating overfitting. As illustrated in Fig 5, DSC begins with a depthwise convolution that processes each input channel independently, followed by a pointwise convolution that fuses these features to produce the final output. This design maintains the multi-scale feature extraction benefits of the Inception module while significantly improving computational efficiency, making it ideal for edge and mobile computing scenarios.

In fabric pilling and fuzzing assessments, using DSC instead of standard convolutions, especially with larger kernels like 3x3 and 5x5, greatly reduces computational complexity and enhances efficiency without compromising feature extraction or classification performance. This approach maintains evaluation precision and real-time responsiveness, offering an efficient solution for applying deep learning in this field. It also demonstrates the flexibility of deep learning in addressing industry-specific challenges.

## Data acquisition and feature extraction

### Data acquisition

**Fig 6:**
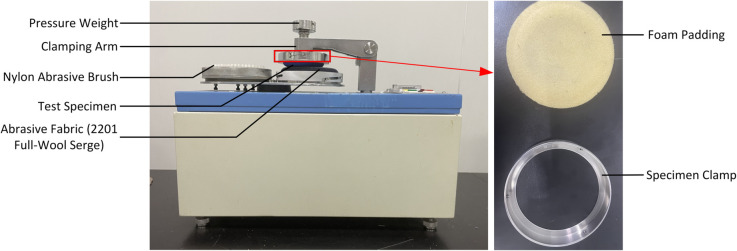
YG502N fabric pilling tester. The YG502N Fabric Pilling Tester used for pilling and fuzzing tests is shown, along with its structural components.

The dataset for this study includes standard pilling images and worn fabric images provided by the Zhejiang Institute of Quality Sciences. Fabric wear tests were conducted following the national standard GB/T 4802.1-2008 “Textiles - Determination of Pilling and Fuzzing - Part 1: Circular Track Method,” using the YG502N fabric pilling tester from Nantong Hongda Experimental Instrument Co., Ltd. Detailed technical specifications of the instrument are listed in Table 1, and the equipment setup is shown in Fig 6.

**Table 1:** YG502N fabric pilling tester parameters.

Parameter	YG502N Fabric Pilling Tester
Power Supply Voltage	AC220V
Operating Power	90W
Current	6A
Frequency	50Hz
Cabinet Rotation Speed	60r/min

**Fig 7:**
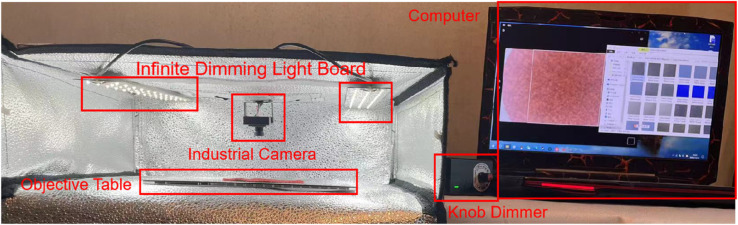
Image acquisition device. The components of the Image Acquisition Device are shown.

A custom-developed image acquisition device was also used to capture high-quality images of worn fabric samples in a controlled darkroom environment, with a stable light source to maintain consistent lighting. Images were captured using a Jieruiweitong DF100-720P industrial CMOS camera, ensuring high-resolution detail capture. A SuteFoto foldable studio and adjustable monochrome light panels were used to comply with the illumination standards of GB/T 4802.1-2008 and ISO 12945-4-2020 [34], enhancing image acquisition accuracy as shown in Fig 7.

During image acquisition, personnel from the Zhejiang Institute of Quality Sciences subjectively rated all samples according to GB/T 4802.1-2008 and ISO 12945-4-2020. The rating criteria are detailed in Table 2, with grades reflecting varying levels of pilling and fuzzing observed on fabric surfaces.

**Table 2:** Pilling and fuzzing grading scheme.

Grade	Description
5	No change.
4	Partially formed pills and/or slight surface fuzzing.
3	Moderate pilling — pills of varying size and density partially covering the specimen surface and/or moderate surface fuzzing.
2	Distinct pilling — pills of varying size and density covering a large proportion of the specimen surface and/or distinct surface fuzzing.
1	Severe pilling — pills of varying size and density covering the whole of the specimen surface and/or dense surface fuzzing.

### Dataset

**Fig 8:**
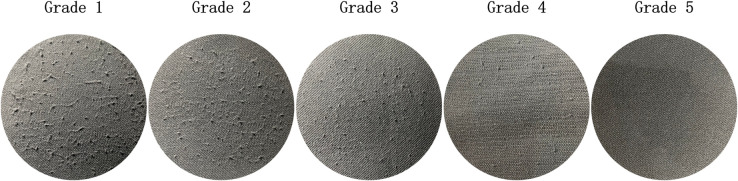
Standard images of wool fabrics. The diagram shows the standard images of wool fabrics provided by Zhejiang Institute of Quality Sciences. These images serve as reference samples for quality assessment and research analysis.

**Fig 9:**
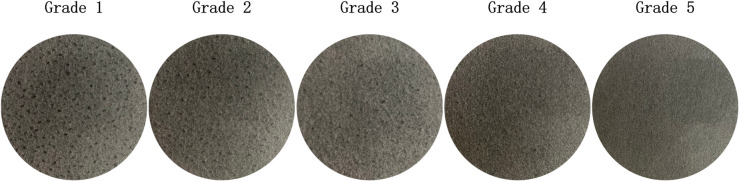
Standard images of cotton blend fabrics. The diagram presents the standard images of cotton blend fabrics provided by Zhejiang Institute of Quality Sciences. These images serve as reference samples for quality assessment and research analysis.

The dataset used in this study includes standard images of Wool Fabric and Cotton Blend Fabric materials provided by the Zhejiang Institute of Quality Sciences, along with additional worn fabric images collected using a custom-developed image acquisition device. It should be emphasized that the model proposed in this study was trained and tested exclusively on Wool and Cotton Blend fabrics, and has not yet been evaluated on other fabric types. Fig 8 shows standard Wool Fabric images, while Fig 9 shows standard Cotton Blend Fabric images.

**Fig 10:**
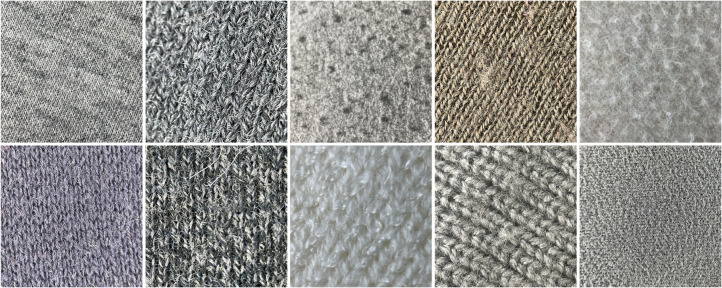
Plain-colored images of wool fabrics. The diagram presents partial plain-colored images of wool fabrics with varying pilling grades. These images are used to assess and compare surface changes in wool fabrics under different wear conditions.

**Fig 11:**
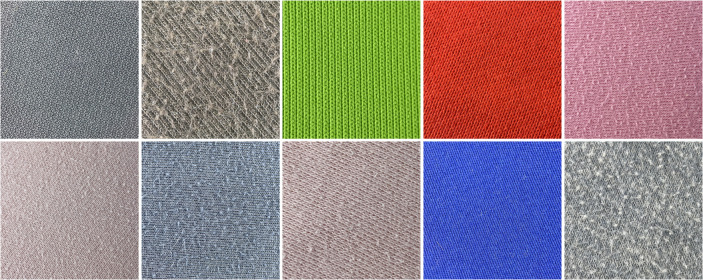
Plain-colored images of cotton blend fabrics. The diagram shows partial plain-colored images of cotton blend fabrics with varying pilling grades. These images are used to assess and compare surface changes in cotton blend fabrics under different wear conditions.

To address data imbalance across pilling grades, we increased testing intensity on the YG502N by extending wear time and pressure, artificially supplementing data for higher wear grades. This yielded a more balanced distribution of images across pilling grades. Figs 10 and 11 show examples of Plain-colored Wool Fabric and Cotton Blend Fabric images collected through this method.

**Fig 12:**
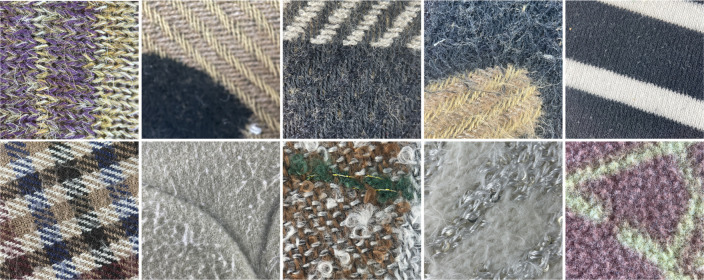
Noisy images of wool fabrics. The diagram presents noisy images of wool fabrics with varying pilling grades. These images, which include noise and artifacts from actual testing, are used to assess and compare surface changes and quality impacts on wool fabrics under different wear conditions.

**Fig 13:**
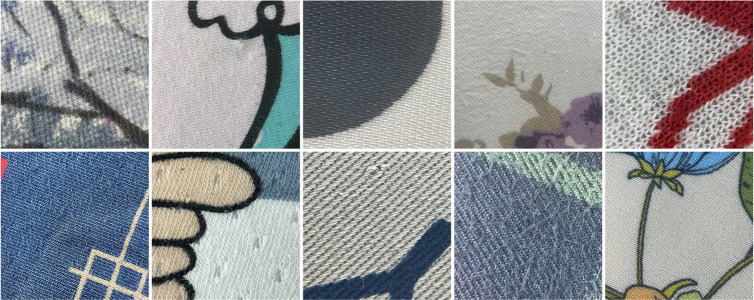
Noisy images of cotton blend fabrics. The diagram shows noisy images of cotton blend fabrics with varying pilling grades. These images, which include noise and artifacts from actual testing, are used to assess and compare surface changes and quality impacts on cotton blend fabrics under different wear conditions.

The initial dataset covered a range of fabrics with inherent patterns, which sometimes obscure pilling features and impact classification accuracy. To improve model robustness and classification accuracy, we collected additional patterned fabric images, adding diversity to the dataset and validating model generalization. Figs 12 and 13 present examples of noisy Wool Fabric and Cotton Blend Fabric images collected for this purpose.

To mitigate the risk of classification bias due to data imbalance, we employed data augmentation techniques to equalize the number of images across different grades and fabric material types. The final dataset contains 38,758 images, as summarized in Table 3. The majority of images were collected from industrial fabric inspections provided by collaborating companies. To protect proprietary information, images involving patented designs or unreleased products were excluded from the public dataset. Furthermore, images containing identifiable information such as company names, product codes, order numbers, or client references were processed by cropping the sensitive regions and standardizing the image format before inclusion. This ensures that the shared dataset maintains confidentiality while supporting reproducible research. The publicly available subset of the dataset, consisting primarily of self-collected worn fabric images, can be accessed at: https://github.com/QIANCALGARY/Fabric-fuzzing-and-pilling-data/tree/master.

**Table 3:** Dataset.

	Cotton Blend		Wool Fabric	
	Plain-colored	Noise	Plain-colored	Noise
Grade 1	1944	1980	1881	1881
Grade 2	1939	1854	1989	1908
Grade 3	1935	2043	1890	1845
Grade 4	1953	1899	1953	1998
Grade 5	2106	2016	1890	1854

### Image preprocessing and feature extraction

This section describes the process of preprocessing raw images to extract grayscale features and the subsequent extraction of Space and frequency domain features. These steps form the foundation for the classification of fabric fuzzing and pilling grades. Using plain-colored Cotton Blend fabrics as an example, we illustrate the impact of image processing at various stages and provide technical details of the methods used.

#### Preprocessing and grayscale feature extraction.

The image acquisition system was designed with cost-effectiveness in mind, using equipment with a high cost-to-performance ratio to enable broader application. However, low-cost devices may struggle with environmental lighting changes and noise. To address this, several preprocessing techniques were applied to enhance image quality while maintaining the cost advantages, improving the accuracy and reliability of subsequent analysis.

Firstly, low-cost cameras are sensitive to environmental lighting variations, leading to uneven illumination. To correct this, we employed the Single Scale Retinex algorithm, which enhances image contrast by correcting for uneven lighting. The principle of this algorithm involves converting the image into the logarithmic domain, applying a Gaussian filter to extract low-frequency information, subtracting the smoothed image from the log-domain image, and then transforming the result back into the Space domain to obtain an enhanced image, as shown in Eq (1).

$$I_{SSR}\left(x,y\right)=e^{\log\left(I\left(x,y\right)\right)-\log\left(G\left(x,y\right)*L\left(x,y\right)\right)},$$ (1)

In the Equation: $I_{SSR}\left(x,y\right)$ represents the processed output image, I(x,y) denotes the input image, G(x,y) represents the Gaussian kernel function, * denotes the convolution operation, L(x,y) signifies the image in the logarithmic domain.

Secondly, to address noise introduced by increased sensitivity, we applied minimum filtering, which is effective for eliminating salt-and-pepper noise and small-scale abnormally bright areas. This technique involves selecting a sliding window and assigning the minimum pixel value within the window to the central pixel. The process is shown in Eq (2).

$$g\left(x,y\right)={min}_{\left(i,j\right)\in W_{x,y}}f\left(i,j\right),$$ (2)

In the Equation: g(x,y) represents the output image, *W*_*x*,*y*_ represents all pixel positions within a window centered at (x,y), f(i,j) denotes the minimum value of all pixel values within the window.

**Fig 14:**
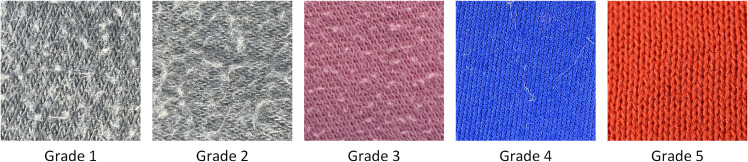
Original image. Shows original images of cotton blend fabrics across five pilling grades. These images provide a visual representation of surface characteristics at different grades.

**Fig 15:**
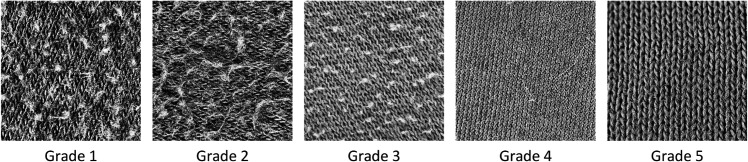
Grayscale feature. Displays grayscale images of cotton blend fabrics across five pilling grades. These images retain important texture and structural features from the original images, supporting noise image classification.

For feature extraction, grayscale conversion was employed to remove color information from the image, simplifying data dimensions and enhancing computational efficiency. Grayscale images retain important features, such as shape, edges, and texture, which are crucial for fabric classification tasks. Fig 14 shows the original image, and Fig 15 presents the preprocessed grayscale image, which retains sufficient texture and structural details.

#### Space domain features.

The next step involves enhancing fabric fuzzing and pilling features in the preprocessed image, followed by binarization to separate pilling from the background. Binarization simplifies the image into two colors, emphasizing fabric fuzzing and pilling while reducing noise, resulting in clearer features. Additionally, binarization reduces the data size, accelerating processing and improving efficiency for feature extraction.

A closing operation, which combines dilation followed by erosion using a structuring element, was applied to remove small noise spots and fill minor gaps. This operation enhances image coherence, smooths object edges, and improves overall quality while preserving the shape of fabric features. The process is shown in Eq (3), with dilation and erosion operations detailed in Eq (4) and Eq (5).

I•B=(I⊕B)⊖B, (3)

$$\left(I⊕B\right)\left(x,y\right)={max}_{\left(i,j\right)\in B}\left\{I\left(x-i,y-j\right)\right\},$$ (4)

$$\left(I⊖B\right)\left(x,y\right)={max}_{\left(i,j\right)\in B}\left\{I\left(x+i,y+j\right)\right\},$$ (5)

In the Equation: *I* is the input image, *B* is the structuring element, (x,y) is the current pixel position being processed, ⊕ denotes the dilation operation, and ⊖ denotes the erosion operation.

**Fig 16:**
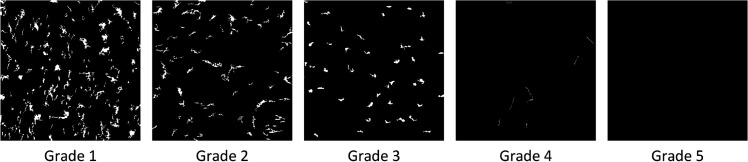
Space domain features. Shows Space domain features of cotton blend fabrics across five pilling grades. Space domain images emphasize the edges of pills, reflecting small-scale structures formed by localized fiber entanglements.

After the closing operation, smaller regions of noise are removed, leaving mainly the contours of fuzzing and fabric texture. Since fabric texture contours generally outnumber pilling contours, we calculated the average area size of the contours, added a 50% offset as a threshold, and applied morphological filtering to remove regions exceeding this threshold. The Space domain features of different fuzzing and pilling grades are shown in Fig 16.

#### Frequency domain features.

Fuzzing and pilling, caused by the entanglement of fibers, create small-scale, irregular structures in the Space domain. These features are represented by point-like or patchy structures, which appear random and lack periodicity. In the frequency domain, such features tend to occupy high-frequency components, while the inherent fabric texture, defined by yarn weaving patterns, occupies the medium to low-frequency range.

To accurately capture the frequency domain characteristics of fuzzing and pilling, we need to convert the preprocessed images to the frequency domain, relying on FFT. Specifically, the Space domain image is transformed into the frequency domain to obtain the frequency domain image, as shown in Eq (6).

$$F\left(u,v\right)=\sum_{x=0}^{M-1}\sum_{y=0}^{N-1}f\left(x,y\right)e^{-j2\pi \left(\frac{ux}{M}+\frac{uy}{N}\right)},$$ (6)

In the Equation: F(u,v) denotes the frequency domain image, f(x,y) denotes the Space domain image, *M* and *N* represent the number of rows and columns of the image, respectively.

**Fig 17:**
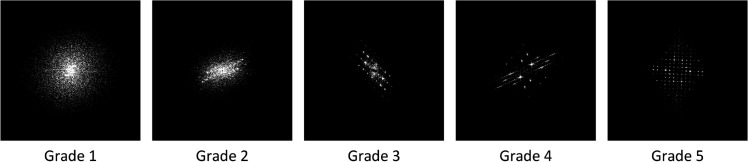
Frequency domain features. Shows frequency domain features of cotton blend fabrics across five pilling grades. Pilling features predominantly occupy high-frequency components, while inherent fabric textures are mainly represented in the mid-to-low frequency range.

Although the Retinex enhancement method and minimum filtering improve image quality, they may introduce high-frequency artifacts and reduce high-frequency details, which could interfere with frequency domain feature extraction. Therefore, we focused on medium to low-frequency components to avoid these issues. Scale transformation techniques were applied to retain only the relevant frequency components, and binarization was used to highlight key frequency features, simplifying the image and making them easier to analyze. The frequency domain features are shown in Fig 17.

In the low-frequency range, fabric texture patterns are clearly visible, but as fuzzing and pilling increase, these features become less distinct. When pilling and fuzzing reach extreme levels, the texture may be obscured by a halo effect due to dense pilling.

#### Resampling and uniform feature image size.

Given the emphasis on medium to low-frequency components during frequency domain feature extraction, the generated frequency domain feature images differ in pixel dimensions from the original color images and Space domain feature images. To ensure consistency in subsequent analysis and compatibility with model inputs, it is necessary to standardize these feature image sizes. For this purpose, we employed resampling techniques aimed at adjusting the resolution of each feature image to a unified specification, matching the input requirements of the classification model. Specifically, we set the target size to 512×512 pixels, a dimension chosen to satisfy the model’s input requirements while optimizing algorithm performance.

Resampling ensures consistency between input images and model requirements, optimizes storage space and computational resources, while maintaining image quality. Among various interpolation algorithms, we chose bicubic interpolation due to its superior performance in handling Space domain and frequency domain features of fabric fuzzing and pilling. Bicubic interpolation facilitates smooth transitions in images, effectively preserving detail and edge clarity, particularly suitable for images containing fine textures. Additionally, bicubic interpolation offers controllable sharpening and smoothing functions, adapting to complex image transformations, ensuring precise reproduction of fabric features at different angles, thereby enhancing both image quality and analysis precision. The specific process of bicubic interpolation is shown in Eq (7).

$$p\left(u,v\right)=\sum_{i=0}^{3}\sum_{j=0}^{3}w_{ij}x_{i}y_{j},$$ (7)

In the Equation: p(u,v) represents the interpolation result at point (u,v), *w*_*ij*_ denotes the weighting coefficient, *x*_*i*_ and *y*_*j*_ are polynomial basis functions dependent on *u* and *v*.

## Experimental results and analysis

### Evaluation metrics

Evaluating model performance based solely on accuracy may not fully capture its effectiveness, especially with imbalanced datasets. In such cases, a model might favor the majority class, overlooking minority class samples. Therefore, multiple metrics are considered.

Accuracy (Acc) is a fundamental metric for model evaluation, prized for its simplicity, ease of calculation, and broad applicability, as shown in Equation Eq (8).

$$Acc=\frac{TP+TN}{TP+TN+FP+FN}\times 100\%,$$ (8)

In the equation: *TP* stands for True Positives, *TN* represents True Negatives, *FP* denotes False Positives, and *FP* signifies False Negatives

Precision (PRC) and Recall (REC) further assess classification performance. Precision measures the proportion of correctly identified positive samples among all predicted positives, as shown in Eq (9). Recall evaluates the proportion of actual positives correctly identified by the model, reflecting its capacity to cover true positives, as shown in Eq (10).

$$PRC=\frac{TP}{TP+FP}\times 100\%,$$ (9)

$$REC=\frac{TP}{TP+FN}\times 100\%,$$ (10)

To address class imbalance, the F1-score combines Precision and Recall as a balanced metric, providing the harmonic mean of these metrics and accounting for both model accuracy and coverage, as shown in Eq (11):

$$F1=2\times \frac{PRC\times REC}{PRC+REC},$$ (11)

The Receiver Operating Characteristic (ROC) Curve highlights the model’s ability to distinguish between positive and negative samples across various thresholds. An optimal ROC curve approaches the upper left corner, demonstrating high recall and minimal false positives. The area under the curve (AUC) provides an aggregate measure, with a higher AUC indicating superior performance.

### Experimental environment and hyperparameter

Experiments were conducted on a dataset consisting of 9,877 images of cotton blend fabrics, targeting the evaluation of pilling and fuzzing severity. The dataset was split into 80% for training and 20% for testing. All experiments were run on a system equipped with an Intel Core i7-6700HQ processor, NVIDIA GeForce GTX 1060 GPU, and 16 GB RAM, using Windows 10, Anaconda3, and TensorFlow 2.7.0 as the software environment.

To determine the optimal training configuration, cross-validation was first employed to explore different combinations of optimizers, learning rates, and dropout rates under the ReLU activation function and a batch size of 32. The Adam optimizer achieved higher classification accuracy with a learning rate of 0.001 and dropout rates of 0.3 and 0.4. In contrast, the SGD optimizer performed best at a learning rate of 0.1 with dropout rates of 0.3, 0.4, and 0.5, as detailed in Table 4.

**Table 4:** Results of cross-validation for parameter optimization.

Optimizer	Learning Rate	Dropout Rate	Acc (%)	FPS
Adam	0.1	0.3	19.58	10.16
		0.4	19.68	10.37
		0.5	19.68	10.00
	0.01	0.3	21.02	9.74
		0.4	21.35	9.88
		0.5	20.89	9.29
	0.001	0.3	85.65	9.83
		0.4	92.01±0.5	9.73
		0.5	21.35	9.77
SGD	0.1	0.3	21.35	9.65
		0.4	66.87	9.76
		0.5	20.85	9.88
	0.01	0.3	88.67	9.36
		0.4	83.26	9.22
		0.5	87.05	9.61
	0.001	0.3	40.87	10.97
		0.4	41.02	10.65
		0.5	40.84	10.53

To further investigate the effects of activation function and batch size, additional cross-validation experiments were performed based on the previously identified optimal settings. These experiments involved testing alternative activation functions, including Leaky ReLU and PReLU, and increasing the batch size to 64. As shown in Table 5, the highest classification accuracy was obtained using the Adam optimizer with a learning rate of 0.001, dropout rate of 0.4, ReLU activation function, and a batch size of 32. The momentum parameters were set to beta1 equal to 0.9 and beta2 equal to 0.999, which helped enhance convergence stability and overall model performance.

**Table 5:** Performance under different training configurations.

Optimizer	Learning Rate	Dropout Rate	Activation Function	Batch Size	Acc (%)	FPS
Adam	0.001	0.3	relu	32	85.65	9.83
				64	86.11	10.16
			leaky_relu	32	83.02	9.18
				64	83.53	9.66
			prelu	32	85.09	8.93
				64	85.78	9.46
		0.4	relu	32	92.01±0.5	9.32
				64	91.46	10.98
			leaky_relu	32	91.60	9.02
				64	91.37	10.78
			prelu	32	90.60	8.23
				64	91.60	9.53
SGD	0.01	0.3	relu	32	88.67	9.36
				64	88.03	10.88
			leaky_relu	32	86.46	9.14
				64	85.99	10.12
			prelu	32	82.08	7.35
				64	80.90	8.66
		0.4	relu	32	83.26	9.22
				64	84.27	10.15
			leaky_relu	32	85.69	9.07
				64	81.07	9.88
			prelu	32	83.16	7.33
				64	83.30	8.00
		0.5	relu	32	87.05	9.61
				64	85.25	10.76
			leaky_relu	32	87.65	8.91
				64	87.95	10.69
			prelu	32	89.35	8.03
				64	89.03	9.14

Although Leaky ReLU and PReLU are theoretically designed to address the “dying ReLU” problem and improve non-linear representation capability, experimental results demonstrated that ReLU consistently outperformed both in this structural image classification task. This is likely due to the nature of structural fabric images, which are characterized by sparsity and clearly defined edges. ReLU’s ability to suppress negative activations helps highlight salient features while minimizing the influence of background noise, a property especially beneficial in this context. Moreover, the absence of additional learnable parameters in ReLU reduces model complexity and risk of overfitting, particularly when used in conjunction with a relatively high dropout rate. Combined with the stable and fast convergence behavior of the Adam optimizer, these advantages led to superior classification performance when using ReLU in this experimental setup.

### Inception module

The Inception module is key to HDAN-PF. Increasing the number of Inception modules enhances expressive power but also raises computational complexity, training time, and overfitting risk. When the number of modules is odd, the computational graph structure better supports parallel processing, improving hardware resource utilization and classification speed.

The optimal number of modules was determined by incrementally adding modules, starting with three, while monitoring performance. A Dropout layer was introduced to prevent overfitting. FPS and accuracy with various module counts are shown in Table 6.

**Table 6:** Performance comparison with different numbers of inception modules.

Number of Inception Modules	FPS	Acc (%)
3	6.86	88.67
5	6.82	89.32
7	4.72	89.37
9	3.20	89.47

As shown, with five Inception modules, classification speed improves by at least 2.1 FPS over deeper architectures, with a minor accuracy decrease of 0.15%. Compared to shallower models, accuracy improves by 0.65% without significant speed loss. Thus, a five-module structure was selected for HDAN-PF.

### Comparative analysis with other classic models

To validate the effectiveness of the proposed HDAN-PF model, a systematic comparison was conducted against two classic convolutional neural network architectures: DenseNet and ResNet. As presented in Table 7, HDAN-PF demonstrates superior overall performance in both inference speed and classification accuracy. Specifically, HDAN-PF significantly improves inference speed compared to DenseNet, while also achieving a 1.06% higher classification accuracy. Although it is slightly slower than ResNet, HDAN-PF surpasses it by 1.77% in accuracy, indicating stronger discriminative capability and model stability, thereby confirming its effectiveness in the fabric pilling grade classification task.

**Table 7:** Classification performance comparison between HDAN-PF and classical CNN models on fabric pilling task.

	ResNet			DenseNet			HDAN-PF		
Grade	PRC	REC	F1	PRC	REC	F1	PRC	REC	F1
1	1.00	0.99	1.00	1.00	1.00	1.00	1.00	0.99	0.99
2	0.99	0.94	0.97	0.92	0.65	0.76	0.96	0.97	0.96
3	0.79	0.83	0.81	1.00	0.92	0.96	0.82	0.85	0.84
4	0.76	0.75	0.76	0.91	0.67	0.77	0.83	0.81	0.82
5	0.99	0.96	0.98	0.87	0.92	0.89	0.99	0.97	0.98
AVG	0.906	0.894	0.904	0.940	0.832	0.876	0.920	0.918	0.918
FPS	7.23			3.29			7.08		
Acc(%)	90.24			88.31			92.01		

To further evaluate the adaptability of the Vision Transformer (ViT) for this specific task, two different input strategies were investigated and compared with the HDAN-PF model. One strategy utilizes the original RGB three-channel color images as input, while the other employs grayscale images fused with multiple frequency-domain and texture-based features. As shown in Table 8, the ViT model achieves a classification accuracy of only 76.73% when using RGB images, which is considerably lower than that of other models. When fused grayscale images are used as input, the accuracy improves to 83.54%, highlighting the positive impact of effective image representations. However, even with enhanced input features, ViT still underperforms compared to HDAN-PF, indicating its limited suitability for this task.

**Table 8:** Performance comparison of ViT under different input strategies and HDAN-PF on fabric pilling classification.

	VIT(RGB)			VIT(hybrid features)			HDAN-PF		
Grade	PRC	REC	F1	PRC	REC	F1	PRC	REC	F1
1	0.64	0.68	0.66	0.74	0.99	0.85	1.00	0.99	0.99
2	0.60	0.63	0.61	0.83	0.61	0.70	0.96	0.97	0.96
3	0.94	0.87	0.90	0.86	0.82	0.84	0.82	0.85	0.84
4	0.89	0.73	0.80	1.00	0.97	0.99	0.83	0.81	0.82
5	0.81	0.92	0.86	0.95	0.95	0.95	0.99	0.97	0.98
AVG	0.776	0.766	0.766	0.876	0.868	0.866	0.920	0.918	0.918
FPS	5.38			4.24			7.08		
Acc(%)	76.73			88.31			92.01		

Further analysis reveals two main limitations contributing to ViT’s performance. First, the ViT architecture primarily captures global structure and color information, whereas fabric pilling grade classification relies heavily on fine-grained local texture and frequency features, which are not adequately represented in standard RGB images. Although using fused grayscale images partially mitigates this limitation, ViT remains less effective due to its insufficient capability in modeling local texture information. Second, ViT typically requires large-scale and diverse pretraining datasets to achieve optimal performance. In this study, however, the dataset is self-collected and relatively limited in scale, which constrains ViT’s ability to fully leverage its architectural strengths.

In conclusion, the HDAN-PF model is better aligned with the task-specific feature requirements and demonstrates stronger generalization and robustness under the current data conditions, outperforming ViT in overall performance. Future research will further explore integrating vision modeling techniques with the rapidly evolving large language models (LLMs) to enable deeper cross-modal feature fusion, advancing intelligent recognition in fabric surface analysis and other complex visual tasks.

### Model optimization and performance enhancement

Although the HDAN-PF model outperforms traditional methods, its accuracy remains at 89.37%, which does not meet the requirements for practical applications. The primary limitation of the HDAN-PF model lies in its constrained ability to extract critical information, making it difficult to fully capture the subtle texture features of fabric pilling and fuzzing. The grading of fabric pilling and fuzzing relies on variations in local fiber structures, such as the density, length distribution, and aggregation of fuzz. However, traditional convolutional operations employ fixed-size receptive fields, which can only focus on local features and struggle to establish long-range dependencies. This limitation affects the model’s performance in handling complex surface texture patterns, particularly when addressing subtle variations across different fabric materials, weaving techniques, and testing conditions. For example, in fabric pilling and fuzzing evaluation, the density, size, and distribution patterns of localized fuzz are interrelated, yet conventional convolutional operations may fail to effectively capture these long-range dependencies. To address this issue, this study introduces a coordinated attention mechanism to enhance the model’s ability to focus on critical features and establish stronger feature associations across Space locations. Specifically, the coordinated attention mechanism is integrated after the initial convolutional layers and before the Flatten layer to assess its optimization effect. Experimental results demonstrate that this optimization significantly improves classification accuracy, as shown in Table 9.

**Table 9:** Impact of CA on performance.

	Before			After		
Grade	PRC	REC	F1	PRC	REC	F1
1	1.00	1.00	1.00	1.00	1.00	1.00
2	0.97	1.00	0.98	0.98	1.00	0.99
3	0.99	0.97	0.98	1.00	0.98	0.99
4	1.00	0.88	0.94	1.00	0.88	0.93
5	0.90	0.99	0.95	0.90	1.00	0.95
AVG	0.972	0.968	0.970	0.976	0.972	0.972
FPS	6.25			6.82		
Parameters	136,302,277			136,237,261		
Acc(%)	96.82			97.07		

As shown in the Table 9, the introduction of the CA mechanism significantly improves the classification accuracy, reaching at least 96.82%. Although the classification speed decreases slightly, the overall performance improves considerably, with a particularly notable increase in accuracy. Placing the CA mechanism before the Flatten layer, rather than immediately after the initial convolutional layer, results in a 0.25% increase in accuracy and an improvement in classification speed. This indicates that the placement of the attention mechanism plays a critical role in enhancing model performance. Consequently, the final implementation positions the CA mechanism after the initial convolutional layer to maximize its benefits. However, the model’s parameter count reaches 136 million, and the classification speed decreases.

This issue primarily arises due to the large number of parameters associated with standard convolution operations. Compared to DSC, standard convolution uses larger kernels and fully connected operations, increasing both the parameter count and computational complexity, thereby impacting the FPS. To further enhance model performance, DSC is introduced to replace standard convolution kernels. By decomposing convolution operations into depthwise convolution and pointwise convolution, DSC effectively reduces both parameter count and computational complexity, thereby improving overall model efficiency while maintaining high performance. The experimental results are presented in Table 10.

**Table 10:** Optimized HDAN-PF Performance.

Grade	PRC	REC	F1
1	1.00	1.00	1.00
2	0.99	1.00	1.00
3	1.00	1.00	1.00
4	0.99	0.91	0.95
5	0.92	0.99	0.96
AVG	0.980	0.980	0.982
Parameters	135,115,512		
FPS	12.079		
Acc(%)	97.92		

**Fig 18:**
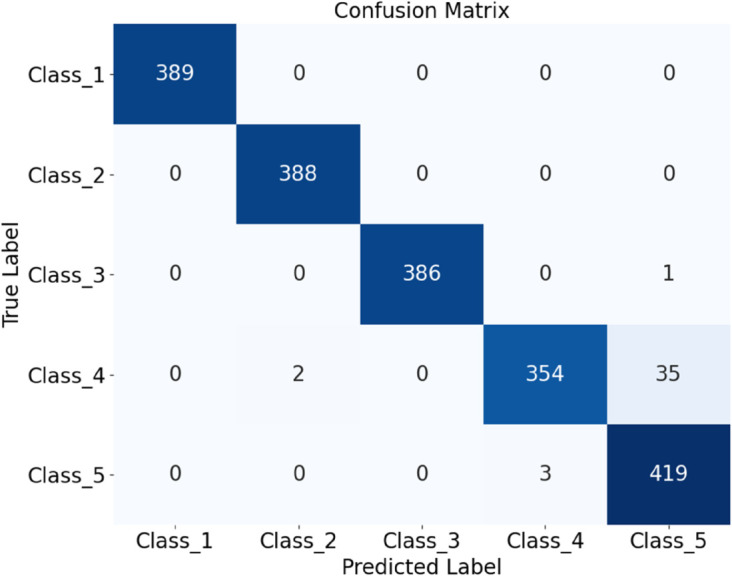
Performance evaluation of the HDAN-PF model with mixed feature fusion: confusion matrix. The diagram presents the classification results of plain-colored images of cotton blend fabrics using the HDAN-PF model. This is the confusion matrix.

**Fig 19:**
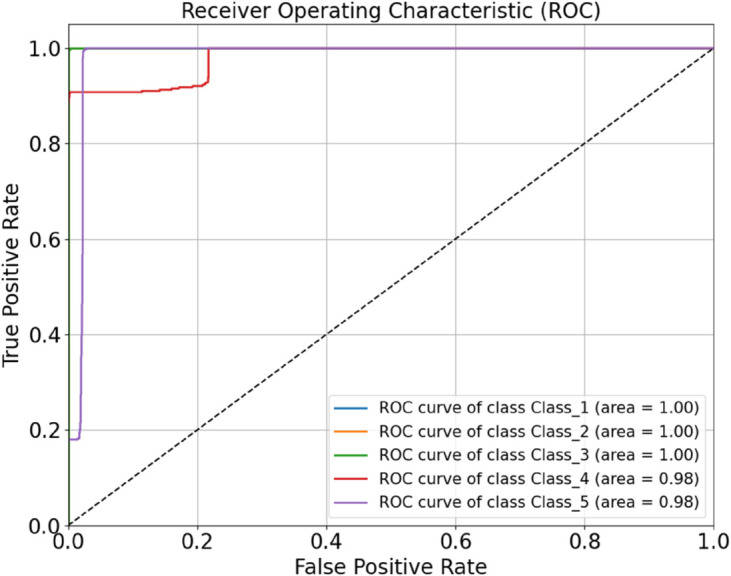
Performance evaluation of the HDAN-PF model with mixed feature fusion: ROC curve. The diagram presents the classification results of plain-colored images of cotton blend fabrics using the HDAN-PF model. This is the ROC curve. Together, they reflect the accuracy and performance of the HDAN-PF model in classifying these images.

As shown in the table, the optimized HDAN-PF model demonstrates outstanding performance, achieving an overall classification accuracy of 97.92%. Compared to the version without DSC, the number of parameters is reduced by 1.12 million, and the classification speed significantly improves to 12.079 FPS, representing a 77.11% increase. Fig 18 presents the confusion matrix of the optimized deep convolutional neural network incorporating hybrid features, illustrating its classification performance under different operating conditions. To further evaluate the model, the ROC curve is plotted, as shown in Fig 19. The AUC values provide a comprehensive assessment of the model’s classification capability across various thresholds.

In the current laboratory workflow, manual evaluation requires 2 to 3 staff members to independently assess the test images, with each sample taking approximately one minute to evaluate. In contrast, the optimized HDAN-PF model achieves a real-time inference speed of 12.079 FPS, resulting in a classification efficiency over 700 times faster than manual assessment. Its accuracy of 97.92% also matches the level of manual evaluation, which achieves 97.82%. Therefore, the model demonstrates excellent real-time performance and stability, making it fully suitable for practical deployment in industrial fabric pilling and fuzzing assessment applications.

The final optimized model contains 135,115,512 parameters and effectively compresses the model complexity while maintaining high performance compared to the initial version. After training, the model size is 327.37 MB, which can be stably deployed on the edge host environment used in this study’s laboratory. During actual operation, the model not only maintains high accuracy but also achieves real-time inference capability, fully meeting the dual requirements of accuracy and responsiveness for industrial fabric pilling and fuzzing assessment. Leveraging the computational resources of the edge host, the model delivers efficient real-time performance and stability, ensuring reliability and operational efficiency in practical applications.

### Ablation study

To evaluate the impact of hybrid features on classification accuracy, seven ablation experiments were conducted to examine the effect of Space domain features, frequency domain features, grayscale image features, and their combinations on classification performance. To ensure the reliability and validity of the experimental results, all experiments were performed under the same conditions with identical training hyperparameters, while maintaining optimization measures such as the attention mechanism and DSC. By comparing the classification accuracy across different experimental groups, the contribution of hybrid features to model performance was comprehensively assessed. The results of the ablation study are presented in Table 11.

**Table 11:** Ablation Study Results.

Group ID	Gray	Frequency	Space	Acc(%)	FPS
1	✓	×	×	79.81	14.120
2	×	✓	×	95.49	15.807
3	×	×	✓	94.18	14.269
4	✓	✓	×	96.62	13.887
5	✓	×	✓	96.23	12.981
6	×	✓	✓	97.44	13.600
7	✓	✓	✓	97.92	12.079

From the data in Table 11, it can be observed that among single-feature models, frequency domain features yield the best overall performance, achieving the highest accuracy of 95.49% while also providing the fastest classification speed at 15.807 FPS. In contrast, the grayscale image feature model, despite having a classification accuracy of 79.81%, retains crucial fabric pattern and texture information that Space and frequency domain features lack. When combined with other features, grayscale features effectively mitigate the negative impact of noise data on accuracy, thereby enhancing classification performance. Although the classification speed of the hybrid feature model decreases slightly, it achieves an accuracy improvement of at least 2.43%, demonstrating that integrating multiple features significantly enhances model accuracy, particularly in handling fabric pattern and texture information with greater stability.

## Discussion and analysis

To assess the generalization and noise resistance of HDAN-PF across various materials and fabric patterns, we conducted a series of tests under diverse conditions. The following sections discuss the model’s noise resistance, performance on different materials, and overall effectiveness on a diversified dataset.

### Individual classification performance under different materials and noise conditions

**Fig 20:**
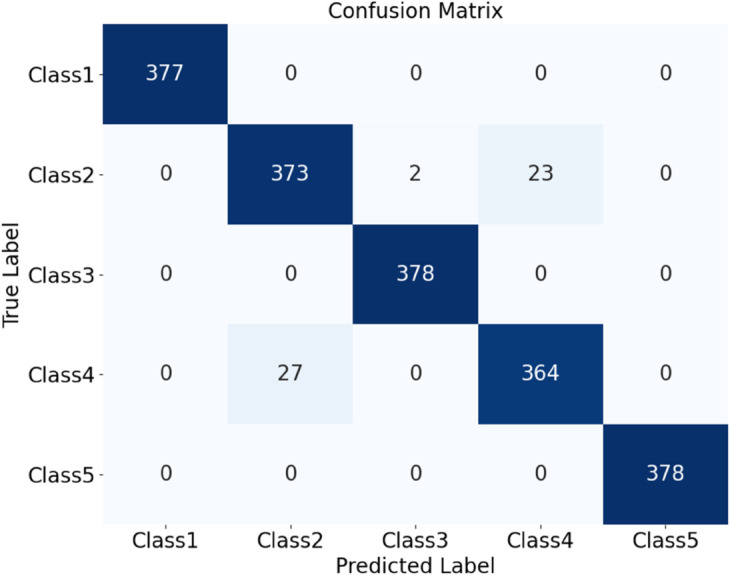
Confusion matrices for classification performance under different materials and noise conditions: noisy wool fabric. The diagram presents the classification results for different types of fabrics using the HDAN-PF model. This is the confusion matrix for noisy wool fabric.

**Fig 21:**
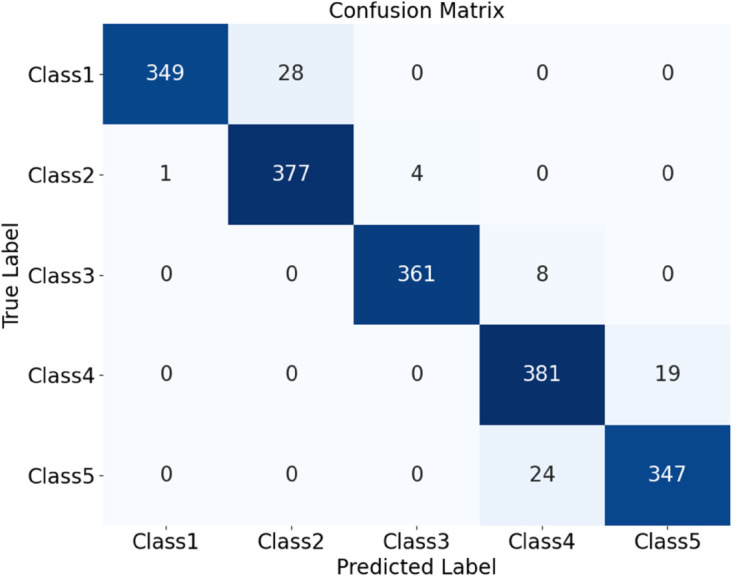
Confusion matrices for classification performance under different materials and noise conditions: noisy cotton blend. The diagram presents the classification results for different types of fabrics using the HDAN-PF model.This is the confusion matrix for noisy cotton blend fabric.

**Fig 22:**
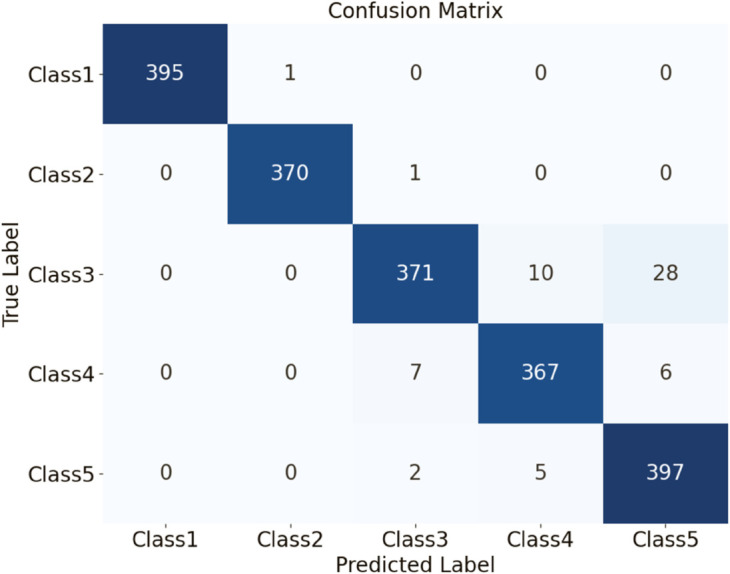
Confusion matrices for classification performance under different materials and noise conditions: plain-colored wool fabric. The diagram presents the classification results for different types of fabrics using the HDAN-PF model.This is the confusion matrix for plain-colored wool fabric. Together, they reflect the accuracy and performance of the HDAN-PF model in classifying images under various materials and noise conditions.

The HDAN-PF model was first tested on datasets comprising plain-colored Wool Fabric, noisy Wool Fabric, and noisy Cotton Blend. Results show that the classification accuracy for noisy Wool Fabric is 93.84%, as shown in Fig 20, and for noisy Cotton Blend, it is 94.79%, as shown in Fig 21. For Plain-colored Wool Fabric, accuracy reaches 97.29%, as shown in Fig 22, and for Plain-colored Cotton Blend, 97.92%, as shown in Fig 18. This indicates a minor accuracy difference of 0.63% between Wool Fabric and Cotton Blend under noise-free conditions, demonstrating strong model generalization across different materials. However, with noise, Wool Fabric accuracy decreases by 3.45%, while Cotton Blend decreases by 3.13%, suggesting that Cotton Blend provides more stable classification performance under noisy conditions and that noise resistance varies slightly by material.

### Comparison of plain-colored and noisy fabrics

**Fig 23:**
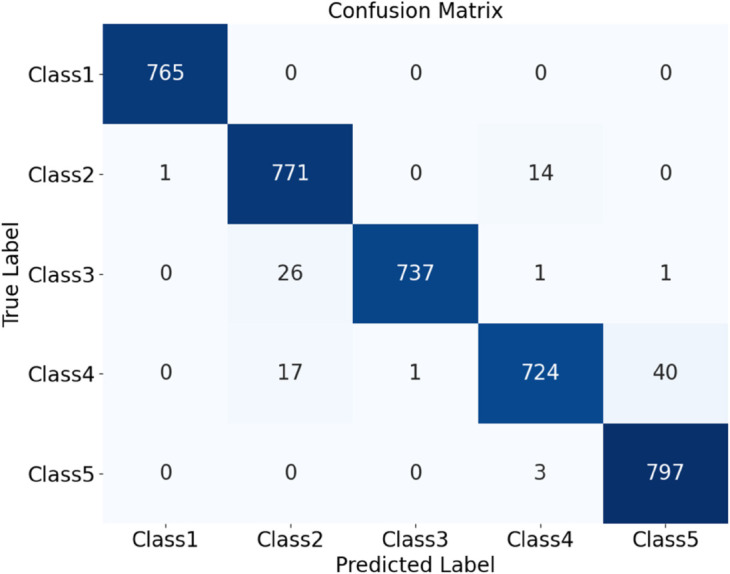
Confusion matrices for classification performance under different materials and noise conditions: all plain-colored. The diagram shows the classification performance of the HDAN-PF model. This is the confusion matrix for all plain-colored fabrics.

**Fig 24:**
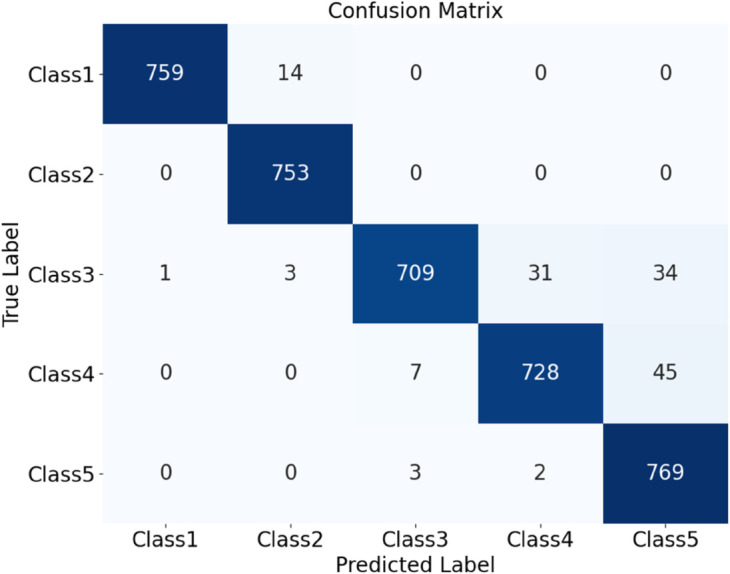
Confusion matrices for classification performance under different materials and noise conditions: (b) All noise. The diagram shows the classification performance of the HDAN-PF model.This is the confusion matrix for all noisy fabrics. Together, they reflect the model’s accuracy and performance across different conditions.

Further experiments compared the model’s performance on plain-colored and noisy datasets of Cotton Blend and Wool Fabric. Results show that classification accuracy for Plain-colored fabrics is 97.33%, as shown in Fig 23, and for noisy fabrics, 96.37%, as shown in Fig 24. Although noise has a slight impact on classification, the model maintains high accuracy across different materials and noise conditions, demonstrating robustness and noise resistance.

### Classification performance on mixed plain-colored and noisy datasets

**Fig 25:**
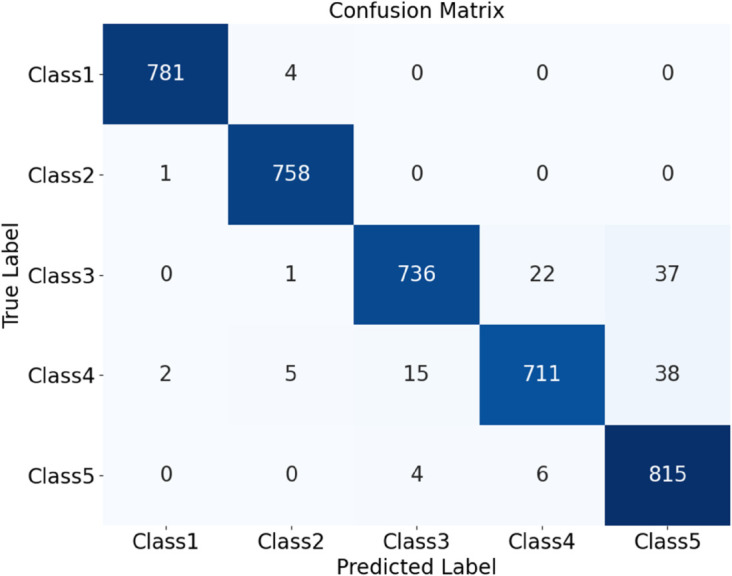
Confusion matrices for classification performance under different materials and noise conditions: all cotton blend. The diagram shows the classification performance of the HDAN-PF model under different material conditions. This is the confusion matrix for all cotton blend fabrics.

**Fig 26:**
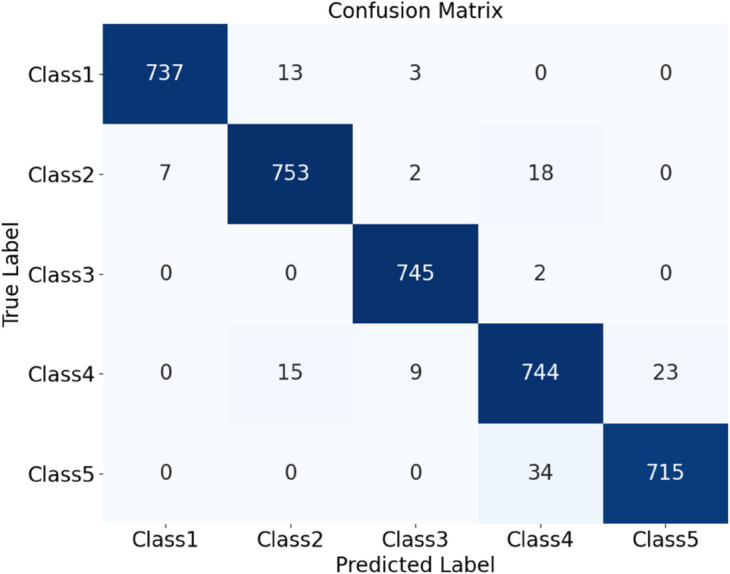
Confusion matrices for classification performance under different materials and noise conditions: all wool fabrics. The diagram shows the classification performance of the HDAN-PF model under different material conditions.This is the confusion matrix for all wool fabrics. These matrices collectively reflect the model’s accuracy and performance in distinguishing between different fabric types.

To further evaluate noise resistance, we tested the model on mixed plain-colored and noisy images of Wool Fabric and Cotton Blend. Results show that the classification accuracy for Wool Fabric is 96.57%, as shown in Fig 25, and for Cotton Blend, 96.70%, as shown in Fig 26. These results confirm that HDAN-PF maintains high classification accuracy on fabrics with noise, underscoring its strong noise resistance.

### Comprehensive test analysis

**Fig 27:**
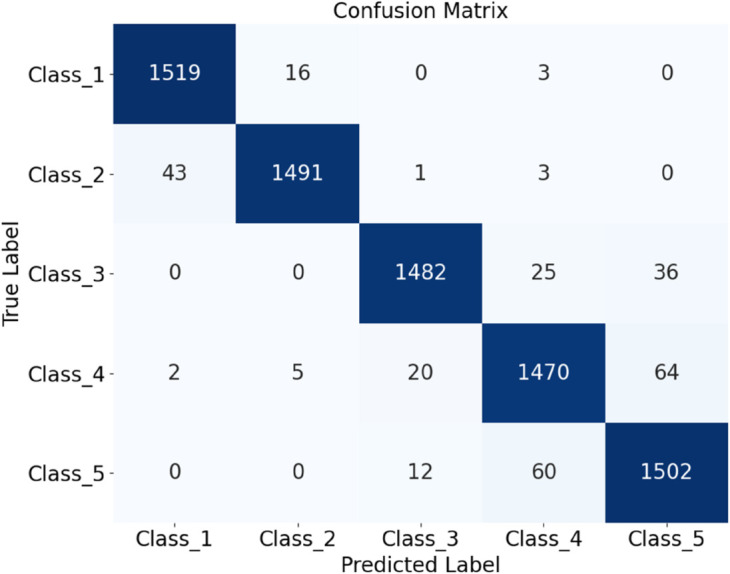
Performance evaluation of the model on multi-material and noisy datasets: confusion matrix. The diagram presents the comprehensive classification results of a mixed dataset including plain-colored wool fabrics, noisy wool fabrics, plain-colored cotton blend fabrics, and noisy cotton blend fabrics using the HDAN-PF model. This is the confusion matrix.

**Fig 28:**
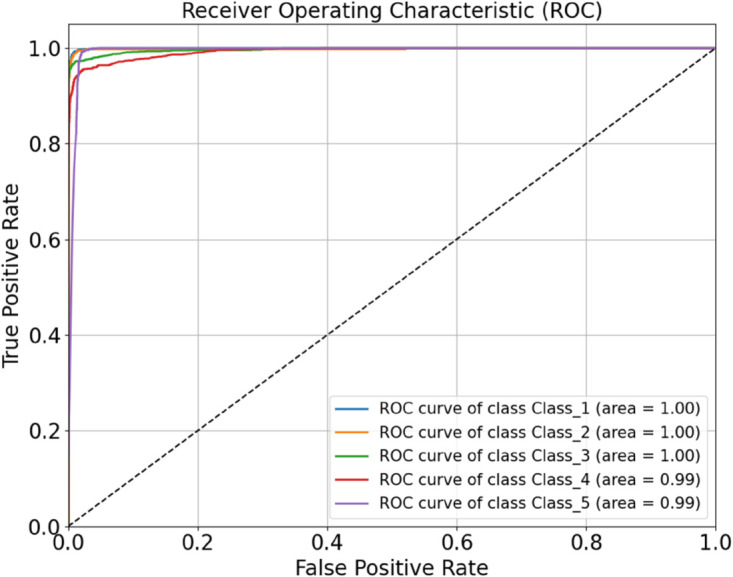
Performance evaluation of the model on multi-material and noisy datasets: ROC curve. The diagram presents the comprehensive classification results of a mixed dataset including plain-colored wool fabrics, noisy wool fabrics, plain-colored cotton blend fabrics, and noisy cotton blend fabrics using the HDAN-PF model.This is the ROC curve. Together, they reflect the accuracy and performance of the HDAN-PF model across various material types and noise conditions, demonstrating its strong generalization capability.

Finally, datasets of plain-colored Wool Fabric, noisy Wool Fabric, plain-colored Cotton Blend, and noisy Cotton Blend were combined for a comprehensive evaluation. The resulting confusion matrix and ROC curve are shown in Fig 27. The model achieved an accuracy of 96.26%, as shown in Fig 28, further demonstrating the applicability of HDAN-PF to both Cotton Blend and Wool Fabric classification tasks. The experiment validates that the model maintains high accuracy in the presence of noise, reinforcing its excellent noise resistance capabilities.

### Statistical analysis

To further validate the reliability and robustness of the proposed model, a statistical analysis was conducted on the experimental results across different datasets. The evaluation metrics included PRC, REC, F1-score, and accuracy. By calculating the mean and standard deviation of each metric, the overall performance and stability of the model under varying conditions were assessed, as summarized in Table 12.

**Table 12:** Statistical Analysis of Model Performance on Different Datasets.

Dataset	PRC	REC	F1	Acc(%)
Plain-colored Wool Fabric	0.9725	0.9727	0.9726	97.29
Plain-colored Cotton Blend	0.9815	0.9791	0.9796	97.92
Noisy Wool Fabric	0.9575	0.9558	0.9562	93.84
Noisy Cotton Blend	0.9699	0.9699	0.9694	94.79
All Plain-colored	0.9743	0.9733	0.9734	97.33
All Noise	0.9652	0.9640	0.9639	96.37
All Wool Fabric	0.9672	0.9657	0.9659	96.57
All Cotton Blend	0.9675	0.9673	0.9674	96.70
All Fabric	0.9630	0.9627	0.9628	96.26
Mean	0.9687	0.9678	0.9679	96.23
Standard Deviation	0.0069	0.0069	0.0068	1.217

The results show that the mean PRC across all datasets was 0.9687 with a standard deviation of 0.0069, indicating consistently high precision. The mean REC was 0.9678 with a standard deviation of 0.0069, suggesting strong recognition capability across different datasets. The mean F1-score was 0.9679 with a standard deviation of 0.0068, reflecting a well-balanced performance between precision and recall. The overall accuracy reached 96.23% with a standard deviation of 1.217%, demonstrating the model’s stability while also highlighting some fluctuation due to the presence of noise in certain datasets.

These findings confirm the robustness of the model. Under various dataset conditions, the model maintained stable PRC, REC, and F1-score values with minimal variation, underscoring its reliability across different fabric types and noise conditions. Moreover, the statistical analysis indicates that despite variations in dataset characteristics, the model consistently achieved high accuracy. This adaptability is particularly valuable in real-world scenarios where textile types and testing environments may vary. The relatively low standard deviations across most metrics further substantiate the model’s stability and practicality for pilling and fuzzing evaluation tasks in the textile industry.

## Conclusion and outlook

This paper presents a hybrid feature-based machine vision approach for assessing textile fabric wear performance, focusing on the objective evaluation of pilling and fuzzing. Using custom-designed equipment, we collected a comprehensive dataset of fabric wear images, including plain-colored color, textured, and patterned fabrics. In the preprocessing stage, we applied a combination of frequency and Space domain enhancements to highlight pilling features, which improved classification accuracy. Additionally, by incorporating texture and pattern features from grayscale images, we enhanced the model’s generalization capability, especially when processing noisy images.

For model selection, after evaluating multiple classic models, we adopted the GoogLeNet architecture and optimized the number of Inception modules through a progressive strategy. The inclusion of CA increased the model’s sensitivity to feature channels and Space positions, enhancing recognition accuracy. Using DSC instead of plain-colored convolutions reduced computational complexity, accelerating the evaluation process while maintaining high precision. These refinements culminated in the HDAN-PF model.The final optimized model contains 135,115,512 parameters, and the trained model file size is 327.37 MB.

Experimental results indicate that this approach achieved a minimum accuracy of 97.29% in assessing wear grades for noise-free, plain-colored cotton blend fabrics; for noisy fabric images, the model’s accuracy reached 96.26%, demonstrating strong noise resistance. Compared to traditional methods relying on subjective inspector evaluations, this approach enhances objectivity and consistency, significantly reducing rating errors caused by human factors and highlighting the advantages of machine vision technology in textile quality inspection.

Currently, due to the collaboration with the Zhejiang Institute of Quality Science, which mainly employs circular trajectory and pilling box methods for testing, this study is limited to specific fabric types (cotton and wool), and the performance on other materials remains uncertain.” “Future work will further optimize the model to assess pilling and fuzzing grades for a broader range of fabric types, enhancing the model’s versatility.

Future research will focus on further optimizing the model architecture to enable wear grade assessment for a broader range of fabric types, thereby enhancing the model’s generalization and adaptability. Although the ViT did not outperform the proposed CNN-based architecture under the current experimental settings—primarily due to limited input feature diversity and a relatively small training dataset—this does not negate its potential under large-scale, multimodal data conditions. We plan to expand the dataset by collecting a wider variety of samples covering different fabric materials (e.g., polyester, silk), weaving techniques (e.g., plain, twill, satin), and post-finishing processes, thereby improving the model’s ability to learn and represent diverse wear patterns.

In addition, to improve the model’s discriminative power and robustness in complex real-world scenarios, we will introduce structured semantic information such as fabric type, weave structure, and processing parameters as auxiliary inputs alongside visual features. A multimodal Transformer-based architecture will be explored to achieve effective fusion of image and semantic information. Notably, related research is already underway in our lab—one of our team members is currently investigating methods to encode fabric attribute information into the Transformer model to enable collaborative text-image-based wear grade prediction. This research direction is expected to enhance the model’s generalization and interpretability, paving the way toward more intelligent and automated textile appearance evaluation systems.

Despite these advancements, there are areas for further improvement. The current model is large and not suitable for deployment on devices with limited computational resourcesTo enable the model to be deployed and applied on devices with more limited computational resources, future research can further lightweight the model through techniques such as quantization, pruning, knowledge distillation, and neural architecture search.
